# Perspectives on enhancing clinical informatics education in the artificial intelligence era

**Published:** 2024-12-04

**Authors:** Hua Min, Xia Jing, Yang Gong, Ping Yu

**Affiliations:** 1 Department of Health Administration and Policy, College of Public Health, George Mason University, Fairfax, Virginia, United States of America; 2 Department of Public Health Sciences, College of Behavioral, Social and Health Sciences, Clemson University, Clemson, South Carolina, United States of America; 3 Department of Clinical and Health Informatics, McWilliams School of Biomedical Informatics, The University of Texas Health Science Center at Houston, Houston, Texas, United States of America; 4 Centre for Digital Health Transformation, School of Computing and Information Technology, University of Wollongong, Wollongong, Australia

**Keywords:** Clinical informatics, Education, Competency, Clinical decision support systems, Generative artificial intelligence, Challenges and opportunities

## Abstract

**Objectives:**

This paper aims to analyze clinical informatic (CI) – a subfield of biomedical and health informatics (BHI) – programs to identify challenges and provide solutions for CI education. Using an online clinical decision support system (CDSS) course as a case study, we demonstrate how these challenges can be addressed. In addition, we discuss the potential impact of generative artificial intelligence (AI), along with the opportunities and risks it presents for CI education.

**Methods:**

This is a perspective paper. The viewpoint analysis is based on a review of formal academic and training programs offered by the American Medical Informatics Association (AMIA) Academic Forum members, Accreditation Council for Graduate Medical Education (ACGME)-accredited CI programs, current literature, and experiences and insights of the authors, who are all CI or BHI educators. An online CDSS course serves as a case study.

**Results:**

We identified the following challenges in CI education: the absence of consensus on CI curriculum content, the diversity of student backgrounds, issues with timely and accurate evaluation of both teaching and learning, insufficient long-term mentoring, and the impact of new AI technologies like generative AI. We used an online CDSS course as an example to demonstrate the solutions in course design, textbook selection, teaching methods, and class project development. These solutions include developing standardized course content for the CI curriculum, incorporating group projects to accommodate diverse student backgrounds, implementing multi-level evaluations, providing ongoing mentoring and support, and cautiously integrating generative AI technologies.

**Conclusions:**

This paper identifies challenges in CI education, shares practical solutions, and discusses the potential impact of generative AI, a double-edged sword for teaching and learning. It provides a foundation and practical reference for CI education, situating it within the broader context of BHI, its foundational discipline. We aim to achieve safer and better healthcare through CI education and practice.

## 1. Introduction

**Figure 1.**
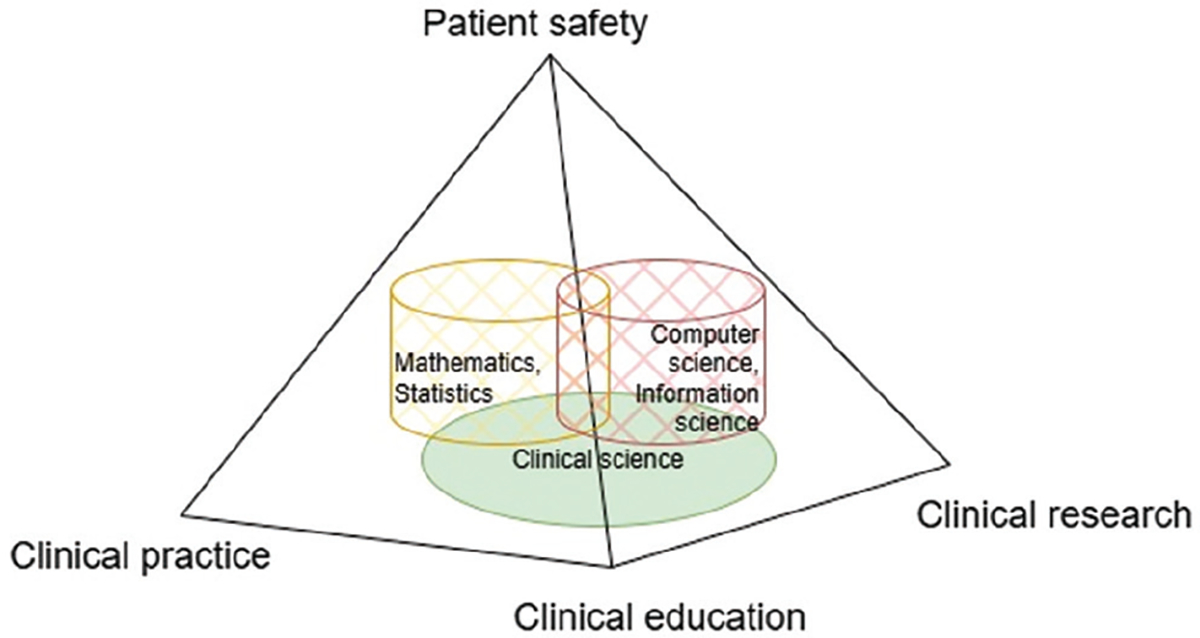
Interdisciplinary clinical informatics and its ultimate goal – patient safety

Clinical informatics (CI), a subdiscipline of health informatics, leverages computer science, information technologies (IT), and statistics to promote safe, efficient, effective, timely, patient-centered, and equitable care during clinical care delivery.^1^ The collaborative and multidisciplinary nature of CI emphasizes the integration of insights from various fields, including medical research, clinical practice, and education, to optimize and innovate healthcare delivery while improving care quality and patient safety. By integrating IT and data-driven insights into clinical workflows, CI aims to minimize errors, ensure informed decision-making, and ultimately enhance the overall quality of patient care processes.^2^ Figure 1 illustrates the interdisciplinary nature of CI and its three foundation dimensions: clinical research, clinical practice, and clinical education. The interaction among these three dimensions is integral for achieving the primary goal of CI – improving patient safety, healthcare quality, and productivity. Therefore, patient safety is presented as the ultimate goal, positioned at the top of the pyramid in Figure 1.

Within the scope of CI, clinical decision support systems (CDSSs) play a pivotal role in promoting patient safety by acting as intelligent tools that assist healthcare professionals in making informed decisions at the point of care. The well-known CDSS framework includes the 5Rs: delivering the right information, to the right person, in the right format, through the right channel, at the right time within the clinical workflow.^3^ By leveraging reasoning approaches and advanced technologies, CDSSs can analyze and prioritize vast amounts of patient data, medical literature, and relevant information in (near) real-time. These systems provide actionable knowledge and evidence-based recommendations, contributing to the optimization of clinical decision-making processes, particularly for diagnosis and treatment.^4–8^ For example, CDSSs assist clinicians in optimizing antibiotic therapy, particularly contributing to antibiotic stewardship protocols focused on addressing sepsis,^5,9^ offering timely alerts and guidance in fall risk management,^7,10^ and assisting in the management of chronic conditions.^11^ With further integration of CDSSs into electronic health record (EHR) systems, and adherence to the 5Rs principles, healthcare providers can deliver higher-quality and safer patient care.

CIs education focuses on training clinical informaticians. Over recent decades, this discipline has advanced to meet the increasing demand for the digital transformation of the healthcare sector.^12^ In 2009, the American Medical Informatics Association (AMIA) published the core content for CI.^2^ In 2017, AMIA further advanced its core competencies for Master’s-level health informatics education, broadening the scope of CI.^13^ These 2017 competencies include example statements of knowledge, skills, and attitudes, organized into 10 functional domains labeled F1 through F10: F1 - Health, F2 - Information Science and Technology, F3 - Social and Behavioral Science, F4 - Health Information Science and Technology, F5 - Human Factors and Socio-technical Systems, F6 - Social and Behavioral Aspects of Health, F7 - Social, Behavioral, and Information Science and Technology Applied to Health, F8 - Professionalism, F9 - Interprofessional Collaborative Practice, and F10 - Leadership. These functional domains form the foundation for Master’s-level CI curriculum development, course accreditation, and quality assessment. Recently, AMIA and the Commission on Accreditation for Health Informatics and Information Management Education (CAHIIM) have begun collaborating to apply these functional domains to undergraduate health informatics education.^14^ Another influence on CI education is the growing public concern about the application of artificial intelligence (AI) in healthcare, particularly its impact on patient safety. This concern has intensified with the emergence of large language models (LLMs) and their visible impact on data analytics and decision-making processes among the general public.^15^

The social-technical and healthcare delivery environment changes have prompted us, a group of experienced CI educators, to reflect on and analyze the challenges and opportunities for advancing our CI education program to better serve our community. Each author has over a decade of experience in CI or biomedical and health informatics (BHI) education, spanning course development, curriculum updates, classroom teaching, and student engagement.

## 2. Research method

In this perspective paper, we adopt a structured analytical approach to examine current trends and challenges within CI education and propose potential strategies for improvement. This approach follows a four-step analytical process.

- Step 1. Review of formal CI/BHI education programs.
  We began by conducting a review of existing BHI educational programs offered by AMIA Academic Forum members. In addition, we analyzed CI programs accredited by the Accreditation Council for Graduate Medical Education (ACGME).^16^ This review allowed us to contextualize the current state of CI education and identify foundational competencies and standards, establishing the groundwork for subsequent analyses.
- Step 2. Analysis of challenges and opportunities in CI education program.
  Building on the program review, we systematically identified and categorized challenges and opportunities within CI education. These insights were derived from an in-depth literature review and direct experiences shared by our team of experienced CI/BHI educators. Through this analysis, we sought to capture the evolving needs of healthcare environments and define the essential skills CI professionals must develop to address these demands effectively.
- Step 3. An illustrative example of an enhanced online tertiary CDSS course
  We developed an online CDSS course to further clarify our insights and proposed solutions. This illustrative example integrates our teaching approaches and is analyzed using the 5Rs framework.^3^ By applying this framework, we examined how these attributes can enhance the design and delivery of CI education, particularly within online and remote learning contexts.
- Step 4. Exploration of emerging technologies.
  Finally, we investigated the potential role of advanced technologies, including next-generation AI and generative AI, in CI education. This exploration involved reviewing recent developments in these fields and analyzing their applicability to CI teaching. We discussed how these technologies can be integrated into CI curricula to enhance educational delivery and provide learners with cutting-edge tools and insights, better preparing them for the evolving demands of healthcare informatics.

## 3. Results

### 3.1. Review of formal BHIs/CIs education programs from the AMIA Academic Forum members and ACGME

The AMIA is the largest international and professional organization for medical informatics. The AMIA Academic Forum is a membership unit that supports universities offering BHI education and training programs. Member universities within the AMIA Academic Forum offer a wide range of formal academic programs at multiple levels, including Bachelor’s, Master’s, Ph.D., Fellowship, Certificate, and Postdoctoral education programs.

**Figure 2.**
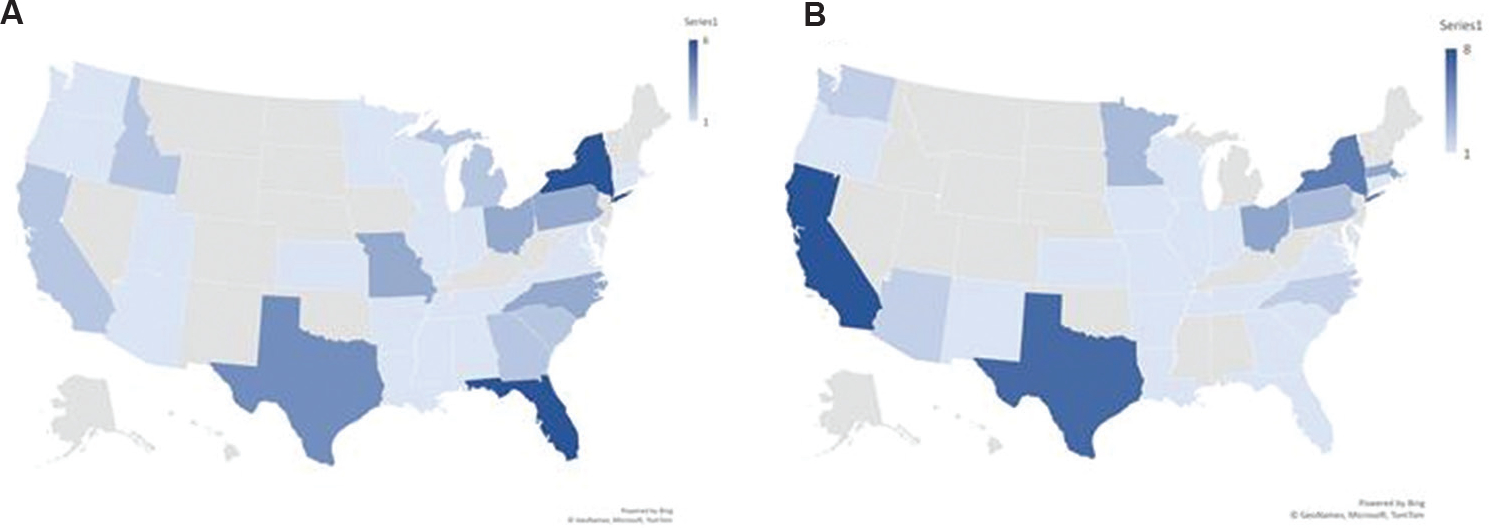
Distribution of biomedical and health informatics (BHI) and clinical informatics (CI) programs in the United States of America. (A) The BHI programs offered by the American Medical Informatics Association (AMIA) Academic Forum members. (B) The CI programs accredited by the Accreditation Council for Graduate Medical Education (ACGME). Note: Darker blue represents a higher number of universities.

In this paper, we review the typical formal education programs, i.e., Bachelor’s, Master’s, and Ph.D. programs, as their quality is highly dependent on curriculum design. Table 1 presents the BHI programs offered by universities, along with their delivery formats, program topics, and objectives. Across the United States of America (USA), 53 universities located in 30 states offer Bachelor’s, Master’s, and Ph.D. programs. Among these institutions, George Mason University, Arizona State University, and the University of Washington provide formal education across all three levels (Bachelor’s, Master’s, and Ph.D.). Fifteen universities offer both Master’s and Ph.D. programs, while three universities – University of Cincinnati, University of North Carolina at Chapel Hill, and UCLA Health - David Geffen School of Medicine – offer only Ph.D. programs. The University of Minnesota School of Nursing and the University of Utah offer Nursing Informatics programs, while other institutions provide programs in health informatics, biomedical informatics, or health information management. Florida, New York, and Texas host the most universities offering BHI programs (see Figure 2A). The curriculum for these programs covers topics such as healthcare, health information technology (HIT), leadership, and practicum (Table 1), all within the 10 foundational domains established by AMIA, as mentioned above. Foundational courses and electives include subjects such as health information systems, information security, data science, computer programming, databases, data mining, data analytics, project management, epidemiology, public health, and leadership.

Meanwhile, the ACGME^16^-accredited CI programs in several medical specialties, including family medicine (8 programs), internal medicine (32 programs), pathology (10 programs), and pediatrics (11 programs). Table 2 provides an overview of these programs. Figure 2B shows that California, Taxes, and New York host the highest number of ACGME-accredited CI programs.

### 3.2. Challenges in CIs education

The CI education program faces a multitude of challenges, including but not limited to:

- Challenge 1: Absence of consensus on course content or modules to meet the competency expectations for the CI curriculum^13^
  The primary challenge in CI education is the absence of consensus on course content or modules that align with the competency expectations for the CI curriculum. Identifying key elements and achieving consensus that aligns with the specific needs and advancements in CI is crucial for developing a comprehensive CI education curriculum. A core competency list and framework have been developed for CI.^2,13,17,18^ However, a consensus on curriculum implementation has yet to be reached due to the broad scope of CI.
- Challenge 2: Diverse student backgrounds and their varied educational needs
  The second challenge in CI education pertains to the diverse backgrounds of students, including professionals such as physicians, nurses, pharmacists, and informaticians.^19^ Some have prior technical experience and capacities, and others bring in prior research, clinical care, and operation experiences in healthcare settings. This diversity adds a multifaceted layer of complexity to educational delivery, as each profession contributes unique perspectives, prior knowledge, and skill sets to the learning environment. This diversity poses challenges in content development, delivery methods, and evaluation of curriculum design and delivery, which must be customized to accommodate the varied backgrounds.
- Challenge 3: Accurate and comprehensive evaluation of the CI curriculum
  The third challenge involves developing precise and comprehensive evaluation/assessment criteria and methods for CI education that align closely with core competencies. Current evaluation techniques can be broadly categorized into two levels:
  - Program-level evaluations, which assess the program as a whole.
  - Course-level evaluations, which include professor’s self-assessment, curriculum reviews, assessment of student work, student surveys, and peer reviews.
- Challenge 4: Provision of continuous mentoring to students beyond the classroom or online sessions^20^
  The fourth challenge is the provision of continuous mentoring to students after the course has ended. Mentoring students is a vital component of CI education, especially relevant for research-oriented graduate students, including Ph.D. students, research Master’s students, and postdoctoral trainees. Effective mentoring involves guiding and advising students through various stages of their research project development, from problem conceptualization within the context of the current status of a discipline to research design, implementation, dissemination, publication, and life-long professional development. Providing student-centered, tailored, rigorous mentoring beyond the classroom for research-oriented graduate students remains an ongoing challenge in CI education. Peer mentoring, often an extension of formal mentoring, is another critical component for professional development in the CI field.
- Challenge 5: The impact of the rapid development of emerging technologies, including generative AI
  Another noteworthy challenge pertains to the discernible impact of generative AI technologies, notably tools like Chat Generative Pre-Trained Transformer (ChatGPT), on the design, implementation, and evaluation of CI education. The impact of AI in education has sparked ongoing debates, with perspectives ranging from its potential benefits to concerns about its drawbacks.^21^ Striking a balance between leveraging AI’s advantages and addressing its challenges in education remains a complex and evolving discourse. As AI technologies and their applications are still in their early stages, comprehensive and systematic development and evaluation are required to clarify their role in medical informatics, and, by extension, in CI education.^22^

**Figure 3.**
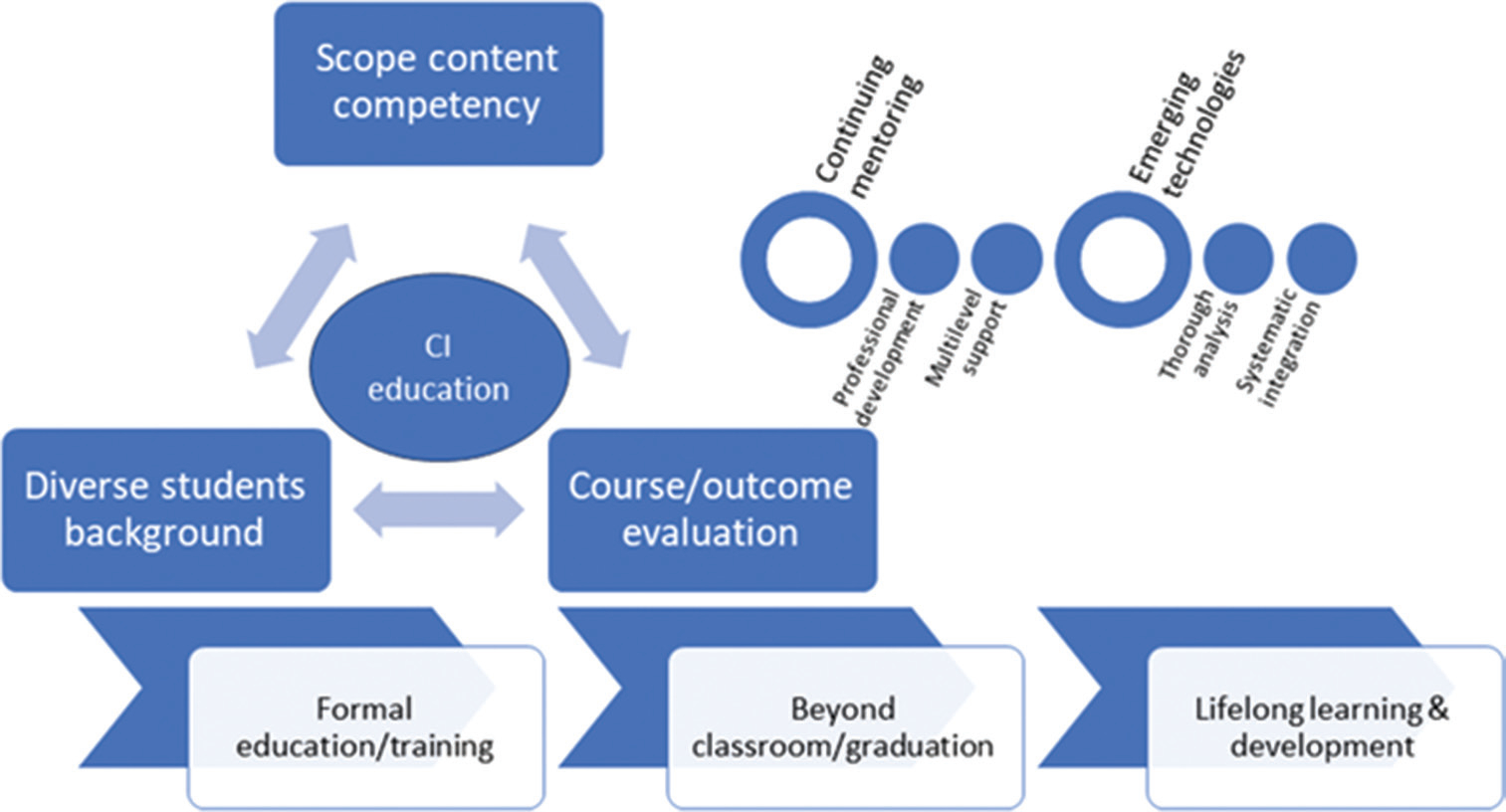
Clinical informatics (CI) education life cycle, challenges, and lifelong learning and development

Figure 3 summarizes the flow of education and lifelong learning, highlighting the various challenges associated with CI education.

We presented our perspective on these challenges at the 34^th^ Medical Informatics Europe Conference (MIE2024)^23^ and will provide a detailed presentation of our proposed solutions using a CDSS course as an example in this paper.

### 3.3. Case study: An open CDSS course

#### 3.3.1. Background of the CDSS course

CDSSs are crucial in CI education as they align directly with AMIA domains, including F2 (Information Science and Technology), F4 (Health Information Science and Technology), and F5 (Human Factors and Sociotechnical Systems). CDSSs also consider the impact of social and behavioral factors and social determinants on decision-making processes (F7: Social, Behavioral, and Information Science and Technology Applied to Health) and emphasize the importance of teamwork and collaboration among various healthcare professionals when utilizing CDSS (F9: Interprofessional Collaborative Practice). CDSS also acts as an effective teaching tool^4,6^ and can help reduce teaching costs.^24^ Existing literature primarily concentrates on using CDSSs to teach medical students and residents about disease diagnosis, treatment, and management. One report highlights the importance of integrating CDSS and e-learning resources into educational curricula and continuous professional development programs, emphasizing the need to tailor these resources to address learners’ knowledge gaps.^25^ However, to date, there has been limited discussion about the challenges, opportunities, and best practices for implementing CDSS courses within the CI educational context. Therefore, we use CDSS as an example to share our teaching experiences on CDSS across multiple universities and to present our strategies for addressing the challenges identified earlier.

CDSS courses aim to equip students with the knowledge and skills necessary to design, implement, utilize, maintain, and evaluate decision support systems and their components effectively. These courses bridge the gap between clinical expertise and technological innovation, providing students with a comprehensive understanding of how computational tools can be utilized in clinical care to enhance decision-making processes in healthcare settings. Typical CDSS course content covers system architecture, CDSS rules, standards, evaluation, dissemination, governance, and ethical and legal considerations.

#### 3.3.2. Major textbooks for the CDSS course

High-quality textbooks are crucial for shaping the curriculum and enhancing the learning experience in CDSS courses. Two widely adopted CDSS textbooks in the field are:

- CDSS*s: Theory and Practice* (3^rd^ ed.) by Eta S. Berner:^26^ This book provides a comprehensive overview of the theoretical and practical aspects of CDSS, encompassing topics such as development, evaluation, and applications.
- *Clinical Decision Support: The Road to Broad Adoption* (3^rd^ ed.) by Robert Greenes and Guilherme Del Fiol:^27^ This book delves into the journey toward widespread CDSS adoption, addressing technical aspects and offering insights into the challenges faced along the way.

In addition, *The Biomedical Informatics: Computer Applications in Health Care and Biomedicine* (5^th^ ed.) by Edward H. Shortliffe, James J. Cimino, and Michael F. Chiang^28^ covers a broader spectrum of biomedical informatics topics, including CDSS. This foundational textbook, broadly adopted in biomedical informatics education, provides significant sections on CDSS and its integration into healthcare while providing a comprehensive coverage of the broader landscape of biomedical informatics. Moreover, both *Health Informatics: Practical Guide* by Katie Fultz Hollis and William R. Hersh (8^th^ ed.)^29^ and *Guide to Health Informatics* (3^rd^ ed.) by Enrico Coiera^30^ include chapters on CDSS.

#### 3.3.3. Main topics covered in the CDSS course

Although decision-making has been repeatedly identified as a core domain of CI,^2,13,17,18^ not all subtopics are agreed upon by stakeholders (Challenge 1). Despite several CDSS and biomedical informatic textbooks containing chapters on CDSSs, none is suitable as the sole textbook for a comprehensive 3-credit CDSS course. Therefore, we developed our open CDSS course based on our teaching experience, existing textbooks, clinical care experience, and AMIA requirements. The resulting open CDSS course includes the following topics: CDSS rules, architecture, standards and terminology, CDSS implementation, evaluation and dissemination, governance, ethics, and legal perspectives from the USA and Europe.

#### 3.3.4. Practice for meeting the diverse needs of students in the CDSS course

As highlighted in Challenge 2, students in a CDSS course often come from diverse professional and educational backgrounds. This diversity includes individuals from healthcare and informatics domains, with varying levels of expertise. To address these differences, we use a pre-course student survey (Appendix A1 for a sample) to collect information about students’ backgrounds and their expectations for the course. One author conducted a student background survey at both the undergraduate and Master’s levels at a public university in the USA. The survey findings show that most undergraduate students (19/22, 86.4%) were from health informatics programs, while 3/22 (13.6%) came from community health programs with a minor in HIT. In addition, one health informatics student had a minor in Computational & Data Sciences, and two others had minors in Psychology.

At the Master’s level, students came from a variety of fields. In one online Master’s class (Table 3, first column), students had backgrounds in pharmacy (14/33, 42.4%), dentistry (4/33, 12.1%), and health informatics (4/33, 12.1%), with others from clinical medicine, nutrition, radiology, nursing, and psychology. In an in-person Master’s class, student backgrounds were similarly diverse, with 20 (37.0%) from engineering, 7 (13.0%) from computer science, and others from pharmacy, dental, health informatics, medicine, public health, biology, business, and nursing. The high percentage of engineering students was attributed to the strong Data Analytics Engineering program offered by the School of Engineering (Table 3, second column). The diverse backgrounds of students require the design and teaching of a CDSS course to be tailored to meet the varying needs and proficiency levels of the student population. This may involve using inclusive teaching strategies, offering diverse learning materials, and providing targeted support for different healthcare professions. For example, bridge or elective courses can address the needs of specific student groups.

Hands-on experience is essential to CI education due to its multifaceted benefits. A recurring request from student surveys is the inclusion of discussions on real-world applications in natural EHR systems or project work. Active engagement through hands-on exercises generates interest and fosters participation. It contributes to experiential learning, enhances problem-solving abilities, and supports social skill development through collaboration. Furthermore, these practical experiences simulate real-world responsibilities, preparing students for professional roles and cultivating adaptability. Through these hands-on endeavors, students can enhance their understanding of CDSS principles and bridge the gap between theory and practice.

Collaboration and teamwork are effective strategies to promote peer learning and bring together students with diverse backgrounds. They are valuable approaches to addressing Challenge 2. Educators can leverage background information collected from pre-course student surveys to customize course content and teaching approaches as one solution to meet diverse students’ expectations. For example, providing assignment options that cater to students’ different skill levels can enhance their learning experience. In addition, instructors can encourage students to share experiences during lectures and discussions and form teams for projects based on their professional backgrounds. Instructors can also design team projects derived from real-world use cases and require hands-on activities that align with industry practices, enabling students to develop familiarity with informatics tools commonly used in healthcare informatics.

By intentionally forming teams that include members with different and complementary professional backgrounds, collaborative projects harness the unique expertise of each student, fostering peer-to-peer learning and enabling students to share knowledge and learn from diverse experiences. Therefore, these collaborative projects not only promote teamwork and effective communication but also align with the interdisciplinary nature of CI.

In the CDSS course, we prepared multiple team-based projects. Appendix A2 presents one example: CDSS rule development and testing. Appendix A3 presents another hands-on project using an open-source EHR for CDSS rule modification. This project is designed to enable students to customize CDSS rules, ensuring clinicians receive tailored information for specific workflows. Hands-on experience in CDSS rule modification not only prepares CI students and trainees to familiarize themselves with the platform’s features but also builds their expertise and understanding of healthcare organization’s clinical policies, procedures, and compliance with relevant regulations and standards.

#### 3.3.5. Practices in course evaluation for the CDSS course

As mentioned in Challenge 3, evaluation is categorized at the program and course levels. The assessment of a CI program’s quality is usually conducted by national or international accreditation committees, such as ACGME,^31^ CAHIIM,^32^ the Healthcare Information and Management Systems Society (HIMSS),^33^ the AMIA Health Informatics Certification (AHIC),^34^ the Australasian Institute of Digital Health Informatician Australasia (CHIA) certification,^35^ and the International Medical Informatics Association (IMIA).^36^ Program-level evaluations assess the curriculum to ensure compliance with AMIA’s 10 foundational domains.

Course evaluation is multifaceted, focusing on both teaching and learning. The quality of a CDSS course can be assessed from four distinct perspectives: (i) educator self-assessment, (ii) student reviews and evaluation, (iii) educator peer reviews, and (iv) evaluation of student learning in the course. This approach ensures a comprehensive evaluation that considers the insights and feedback from educators, students, and colleagues while providing a comprehensive evaluation of teaching and student learning outcomes.

Self-assessment for a CDSS course involves educators evaluating their teaching methods, course content, and overall instructional practices. The self-assessment includes the following components:

- Teaching methods: Evaluating the use of lectures, hands-on activities, case studies, and other approaches to convey CDSS principles to students.
- Curriculum reviews: Ensuring the curriculum addresses core competencies and skills
- Currency of the CDSS course content: Keeping the CDSS course aligned with the latest advancements and trends, such as AI in CDSS.
- Student engagement: Assessing the level of student participation and interaction during CDSS classes
- Multi-level assessment of student work: Evaluating student understanding of CDSS through various means, including, but not limited to, open or closed exams, quizzes, oral or poster presentations (individual or group), writing assignments, project designs, group or individual projects, and student peer-assessment for group projects.

Student surveys are a commonly used tool for course evaluation, conducted at three stages: before (pre-course), during (mid-course), and after (end-course) the course. Pre-course assessments enable instructors to understand students’ prior knowledge, experience with CDSS and related topics, and their expectations. Based on this knowledge, educators can design tailored teaching content. Mid-course assessments collect progress data, allowing instructors to make timely adjustments to teaching strategies. End-course evaluations measure overall student achievement, including theoretical and practical understanding of CDSS principles, and assess the quality of hands-on projects. These assessments ensure continuous improvement and alignment between teaching preparation and student needs. In addition, informal student feedback can be obtained through open-ended questions at the end of each class, such as “What are the muddy points from today’s class?” or “Do you have any questions about today’s topic?” Feedback from these open-ended questions allows instructors and course designers to adjust course content promptly.

Peer evaluation focuses on pedagogical techniques, communication, and alignment with learning objectives. It provides a more balanced and unbiased perspective compared to student evaluations, which can sometimes be influenced by personal biases.^37^ Peers, fellow educators with expertise in CDSS, or educational researchers (typically serving as a university-wide support resource to enhance teaching in higher education across disciplines) offer constructive feedback on teaching methods, delivery, course content, and overall instructional strategies. This collaborative process helps CDSS educators improve their teaching practices, pinpoint areas for enhancement, and integrate diverse perspectives into teaching methodologies. Moreover, peer evaluation contributes to a culture of continuous improvement in academic institutions, fostering continuous teaching excellence and elevating the overall quality of education.

Rubrics provide a clear and structured framework for evaluating student work, including exams, quizzes, projects, and presentations, based on predefined criteria and performance levels. By using rubrics, educators can ensure that their assessments are consistent, fair, and transparent. Students also benefit from rubrics by understanding expectations, guiding their efforts, and improving their learning outcomes. Appendices A4 and A5 present sample rubrics for project peer evaluation and project assessment.

#### 3.3.6. Practices on continuous mentoring

Mentoring in CI is a key to developing leaders in research ethics, CI knowledge and expertise, critical thinking, quantitative and qualitative data analytics, and IT skills. Mentors not only guide students’ and trainees’ research and professional development but also serve as role models for mentees’ career advancement. With ongoing support and adaptable strategies, strong mentor–mentee relationships are essential for fostering lifelong learning and continuous development in this dynamic field.

To keep pace with advancements in medicine and healthcare, lifelong learning and continuing professional development are required for healthcare professionals, including those in CI. Therefore, CI students should cultivate a mindset of continuous growth and development through their career trajectory, seeking guidance and support even after graduation. During their studies, students usually receive structured guidance from professors and supervisors, along with peer support from their cohort. However, it is essential to adopt practices that ensure the continuity of support for CI program graduates. For example, engaging in professional mentoring through professional organizations and programs, maintaining contact with former professors, mentors, and supervisors, and pursuing diverse learning avenues (such as self-learning and professional coaching) can help individuals acquire new skills and advance their careers. Peer mentoring is another valuable strategy that should be promoted to ensure the continuous professional success of CI learners.

#### 3.3.7. Practices in adopting emerging and evolving AI technologies

AI is an emerging technology transforming both clinical practice and education. This transformation is driven by the increasing integration of AI technologies into clinical practice.^4,15,21,38–40^ The introduction of LLMs has brought new paradigms for data analytics, model development, and decision-making.^41–45^ In the CDSS application, ChatGPT performs well in certain types of CDSSs, such as knowledge-based, alerting, diagnostic, therapy planning, and workflow systems, but struggles with those requiring complex data analysis, real-time data processing, or advanced machine learning algorithms.^46^

AI holds promise in CI education, particularly in enhancing activities like information retrieval from online resources, the generation of summary reports, personalized learning, the use of interactive learning tools, and the improvement of patient–provider communication through platforms such as ChatGPT.^38^ AI can assist in developing personalized learning pathways by adjusting to each student’s pace and prior knowledge. For example, ChatGPT can offer tailored explanations and resources based on students’ backgrounds, allowing learners from diverse disciplines and experiences to address gaps in their CDSS knowledge. This approach is especially beneficial in diverse classrooms, where students may have varying levels of familiarity with CDSS concepts.

Generative AI can also create dynamic, case-based scenarios that simulate real-world CDSS applications. For instance, students could interact with virtual patient cases where the AI generates different outcomes based on students’ choices, helping them understand decision pathways within CDSS contexts.^47^ This method allows students to experience simulated decision-making processes in knowledge-based, alerting, and diagnostic CDSS, encouraging critical thinking and practical application without the risks associated with real patients.

Generative AI can further enhance learning by creating virtual patients that simulate various medical conditions, providing a safe environment for students to practice CDSS-guide decision-making.^48^ For instance, virtual patients could simulate age-related health concerns, chronic disease management, or acute symptoms that respond to students’ inputs. These simulations illustrate how CDSS tools guide care plans, allowing students to experience how a CDSS informs their decisions. This immersive experience strengthens their skills in navigating clinical information safely.

AI-powered chatbots can provide instant feedback and support, helping students clarify concepts and receive guidance on course materials outside traditional classroom hours. For CDSS education, where students may need assistance understanding complex AI or CI principles, tools like ChatGPT can explain these principles in a simplified and accessible manner.

While research on integrating generative AI into CI education is still limited, insights from broader educational studies indicate substantial potential for these tools to support innovative teaching methods. These studies provide valuable comparisons of the benefits and challenges of generative AI in higher education, offering lessons that CI educators can apply.

Traditional CI teaching methods often focus on didactic approaches, face-to-face interactions, and structured feedback. In contrast, generative AI offers on-demand, personalized assistance, enhancing flexibility and accessibility. A comprehensive study by Bukar *et al*.^49^ explored the integration of ChatGPT into educational practices across multiple disciplines, highlighting both positive impacts and cautionary challenges. Reported benefits include improved student engagement, enhanced learning outcomes, and support for academic inquiry. However, significant concerns remain regarding misinformation, academic integrity, data privacy, and potential biases. To mitigate these risks, Bukar *et al*. emphasized the importance of developing clear institutional policies for the ethical and informed use of AI tools in educational contexts.

In healthcare, where data integrity and accuracy are paramount, the integration of generative AI raises unique concerns. A survey of healthcare professionals in Saudi Arabia^50^ found that 75% were comfortable with integrating ChatGPT into healthcare contexts, particularly for tasks such as medical literature appraisal, patient support, and decision-making assistance. However, respondents identified the need for transparency and rigor in information sources, especially in contexts where the AI guidance could influence clinical decisions. These findings underscore the importance of addressing reliability and ethical concerns, especially in healthcare-focused education, where the implications of AI use can be far-reaching.

Similarly, a case study by Sandu *et al*.^51^ examined the role of generative AI in an Australian higher education setting, supporting the notion of AI as a transformative educational tool. In a data analytics course, students reported an estimated 17% improvement in academic performance when using ChatGPT, noting the tool’s ability to provide personalized, flexible support. Nonetheless, challenges included ChatGPT’s limited understanding of nuanced queries and a reduction in opportunities for human interaction – a critical component of education. This study highlights that while generative AI can enhance student learning experiences, maintaining interpersonal and higher-order cognitive engagement remains essential.

Lastly, an interview study^52^ gathered insights from educators and students in Thailand regarding ChatGPT’s application in education. The tool was appreciated for providing immediate feedback and assisting with routine inquiries, allowing educators to focus on more complex instructional tasks. However, limitations were noted regarding data privacy, ethical issues, and the reduction in direct human interaction. These findings emphasize the need to carefully balance AI assistance with traditional teaching methods to preserve essential educator–student engagement.

## 4. Discussion

### 4.1. Global perspectives on curriculum alignment: Meeting regional and cultural needs in CIs education

A critical aspect of advancing CI education is ensuring that curricula are designed to meet the varying levels of technological development across different countries. Zainal *et al*.^53^ conducted a literature review on existing CI training in medical schools, highlighting the need for curricula to align with a country’s level of healthcare digitization. In less digitally advanced countries, curricula should focus on fundamental CI skills, such as collecting, analyzing, and using health data. In contrast, more digitally transformed countries should prioritize advanced CI skills, including AI, database management, CDSS, health information exchange, and metacognition.^53^ The study also noted a lack of alignment between CI curricula and ACGME CI milestones,^12^ which is essential for preparing students for the rapidly evolving field of clinical care. This alignment is especially important for CDSS education. In digitally advanced countries, CDSS should be a key part of the curriculum to equip students with the skills needed to use these systems effectively.

Collaborative efforts and partnerships are vital for advancing international CDSS education. By collaborating with universities, research institutions, healthcare organizations, and industry partners from different countries, institutions can develop innovative educational programs, share resources and best practices, and remain up to date with industry trends. These collaborations are crucial for providing students with a comprehensive and relevant education in the field of CDSS.

Table 4 summarizes the challenges faced in CI education, our practices for addressing these challenges, and the lessons learned through these practices. While we have proposed strategies and solutions to tackle challenges in CI education, this list does not imply that all challenges can be successfully resolved. Addressing cultural and regional variations in CDSS education is essential to ensuring that the content and delivery of CDSS courses are relevant and effective for diverse student populations. One approach to achieving this is incorporating case studies, examples, and scenarios that reflect students’ cultural and regional contexts, as well as their reasoning processes during clinical decision-making. This effort helps students relate to the material and understand how CDSS concepts apply to their specific healthcare settings. Furthermore, inviting guest speakers or experts from diverse cultural backgrounds can provide unique insights and perspectives on CDSS implementation and use.

### 4.2. Interdisciplinary teamwork in CDSS education

Interdisciplinary teamwork is a fundamental aspect of clinical care. By bringing together professionals from various disciplines, such as physicians, nurses, pharmacists, informaticians, and health administrators, a more holistic approach to patient care can be achieved. Similarly, this interdisciplinary approach is fundamental for CDSS education, where professionals from these fields comprise both the faculty and the student body for the CDSS course. A study from the University of Utah showed that students entering the medical informatics program had diverse educational backgrounds, most commonly in medicine, engineering, computer science, or biology.^19^ Our student background surveys revealed a similar diversity within the student body. Through collaborative learning, students from different backgrounds can develop a comprehensive understanding of CDSS and its role in improving patient safety and healthcare delivery. One of the class projects (Appendix A2) was designed as a collaborative team project, requiring members to come from diverse backgrounds: At least one student with a medical, nursing, or other healthcare-related background, one with a computer science or information systems background, and one with a healthcare administrative background.

### 4.3. The role of AI in clinical practice and education

The studies discussed above suggest that while generative AI offers unique opportunities for enhancing CI education, its integration should be carefully managed to address potential drawbacks and align with traditional educational objectives. We recommend that educational institutions develop comprehensive policies to guide the ethical and practical use of AI in the curriculum. In addition, targeted strategies that combine traditional and AI-supported methods, such as supplementing AI-generated feedback with in-depth instructor interactions, can provide a balanced approach. These strategies promote effective learning outcomes while upholding critical aspects of academic integrity and human-centered education, which are essential in CI training. The implementation of AI in CDSS demands even greater scrutiny to ensure that the quality and reliability of AI-generated outputs benefit patients without unintended detrimental consequences. Evaluating AI-generated CDSS involves validating its accuracy, recall, sensitivity, specificity, and predictive value against established clinical guidelines and real-world patient data. Recommendations generated by AI systems must be clinically relevant, actionable, and aligned with current best practices. Moreover, examining AI systems for biases is crucial to ensure fairness and diversity in training data. AI-based CDSS raises ethical, social, and legal challenges, including concerns about bias, privacy, and patient–physician trust.^54^ If these challenges are not carefully addressed, they could negatively impact patient care and create inequities.

Furthermore, the effective integration of AI tools into curricula requires sufficient faculty training and thorough, systematic testing of these tools. Future studies should address these aspects before AI is systematically incorporated into CI education. In addition, assessing the ultimate effectiveness of integrating AI into CI education necessitates a systematic comparison of students’ learning outcomes achieved through innovative versus traditional teaching methods. Such comparisons could help identify the most effective strategies for integrating AI into CI education while preserving essential human-centered skills. However, this comparison lies beyond the scope of the current paper.

Another key aspect of AI in CDSS education is the integration of interpersonal skills, such as communication, empathy, and active listening, alongside a focus on preventing potential violations of human rights. These skills are critical for CI professionals to build trust with healthcare providers, IT professionals, administrative personnel, vendor representatives, and patients. They also support informed decision-making, foster the adoption of IT technologies in healthcare, and ultimately improve healthcare outcomes. Training in these skills may benefit from interdisciplinary teamwork, role-playing exercises, and case study analyses, which can improve CI professionals’ awareness of and proficiency in these soft skills through targeted practices. Assessing such skills differs from traditional knowledge testing. Instead, these skills should be evaluated in simulated environments using real or hypothetical cases, which demand significant preparation and design on the part of instructors. Meanwhile, experience and practice in nursing and medical education can serve as valuable references. AI can further improve communication skills training through simulated interactions and role-playing, while also identifying ethical dilemmas and guiding discussions on human rights. Given that AI in education is still in an experimental stage, thoughtful integration is crucial to ensure that its incorporation into CDSS enhances decision-making processes and patient care while maintaining the human elements critical to healthcare. In addition, it is important to update AMIA competencies in CI education to reflect the evolving technological landscape and its applications in healthcare.

## 5. Conclusion

In conclusion, interdisciplinary teamwork is crucial for CI education, as showcased by the described CDSS course. This paper highlights the significance of interdisciplinary collaboration among professionals from various backgrounds in CI education, outlines the major challenges faced, proposes solutions, and shares our experiences in this field. We aim for this paper to serve as a valuable reference for peers in the emerging domain of CI education. While AI may play a critical role in CI education, it is still at its early stage. Together, clinical research, practice, and education contribute to creating a safer and more efficient healthcare environment.

## Figures and Tables

**Table 1.** The list of universities from AMIA Academic Forum members offering formal Bachelor’s (BS), Master’s (MS), and PhD programs in applied health informatics

University	State	Program Name	Format	BS	MS	PhD	Topics
George Mason University	Virginia	Health Informatics	On-campus and online	P	P	P	Health information systems, information security, data science, computer programming, health care databases and data mining, data analytics, project management, and process improvement are covered.
Indiana University, School of Informatics and Computing	Indiana	Biomedical Informatics/Bioinformatics/Health Informatics	On-campus and hybrid	P	P		Learn to analyze patient data to deliver precision health care using the most technologically advanced and secure methods.
Arizona State University	Arizona	Biomedical Informatics/Health Informatics	On-campus and online	P	P	P	Acquiring data, data management, knowledge representation, modeling, and machine learning.
University of Washington	Washington	Biomedical & Health Informatics/Clinical Informatics & Patient-Centered Technologies	On-campus	P	P	P	Data science, health & well-being, human-computer interaction, information architecture, information assurance and cybersecurity, information management, information & society, and software development.
Texas State University	Texas	Health Informatics and Data Analytics	Online	P	P		Ensure the availability, quality, integrity, usefulness, and security of health information required for evidence-based clinical decisions, reimbursement, compliance, and healthcare business operations.
University of South Florida	Florida	Health Informatics	Online	P			Understand how people interact with information and technology; the complexities of the information society; information creation, storage, and organization applications and theories; information architecture; and related knowledge and skills needed to design, implement, and evaluate new tools and approaches to solve emerging information problems.
Johns Hopkins University School of Medicine	Maryland	Applied Health Sciences Informatics/Health Sciences Informatics-Research	On-campus and online		P		Lead or support strategic health informatics projects in clinical settings. Analyze clinical data to assess evidence-based practices. Help develop or lead clinical knowledge management tools. Lead or support the development, selection, and implementation of health IT systems.
University of Missouri	Missouri	Biomedical and Health Informatics/Bioinformatics	On-campus and hybrid		P		Design databases, plan clinical trials, and analyze clinical data. Implement electronic health record systems. Research and publish on biomedical informatics in genetics. Collaborate across fields like engineering, computer science, and healthcare. Contribute to advancements in biomedical informatics.
NYU Grossman School of Medicine	New York	Biomedical Informatics	On-campus		P		Molecular signatures and personalized medicine, computational causal discovery methods, biomedical information retrieval and scientometrics, high-throughput assay informatics (next-generation sequencing, proteomics, metabolomics and image analysis), modeling and simulation of biological systems.
Emory University School of Medicine	Georgia	Biomedical Informatics	On-campus		P		Effective use of biomedical data, information, and knowledge for biomedical research. Application of data and knowledge in clinical research. Use of decision support systems to improve human health outcomes.
UTHealth Houston	Texas	Biomedical Informatics/Health Informatics	On-campus and online		P	P	Explore EHRs and CDSS. Study data interpretation and knowledge management. Understand how to collect, process, and transform biomedical data into health information. Gain knowledge in technology assessment, quality improvement, data analytics, and precision medicine.
UTHealth Houston and UT Austin School of Pharmacy	Texas	Biomedical Informatics	Hybrid		P		Examine EHRs and CDSS, focusing on enhancing these tools. Learn data interpretation, knowledge management, and how to transform health and biomedical data into actionable information and knowledge. Understand core clinical informatics disciplines, including technology assessment, quality and outcome improvement, data analytics, and precision medicine.
University of Central Florida	Florida	Health Care Informatics	Online		P		Health care database management, systems analysis and design, privacy and security, and epidemiology and analytics.
East Carolina University	North Carolina	Health Informatics	Hybrid		P		EHRs, health data structures, evaluation methods in health informatics, health information privacy and security, social and organizational issues in health informatics, and consumer health informatics.
University of Michigan Medical School	Michigan	Health Informatics/Information	On-campus		P		Understand diverse needs to ensure health technology promotes equity. Apply health informatics ethically to solve health issues. Use behavioral and social science theories to design interventions. Develop solutions tailored to healthcare contexts. Implement innovations with awareness of history, culture, and policy to reduce disparities. Evaluate interventions to measure impact and prevent inequities. Communicate and collaborate effectively to address informatics challenges.
Grand Valley State University	Michigan	Health Informatics and Bioinformatics	On-campus		P		Programming: R, Python, Unix. Data: management, mining, integration, analytics, DNA sequencing. Fields: biology, bioinformatics, oncology, computer science, mathematics. Skills: research, creativity, operations, IT, communication, presentations, management.
Louisiana Tech University	Louisiana	Health Informatics and Information Management	Online		P		Healthcare compliance, database architecture, and medical vocabulary systems. Advanced statistical methods, health information security, and introduction to health informatics. Project management, evaluation of information systems, and consumer health informatics. EHR infrastructure, healthcare information analysis, and leadership in healthcare.
Nova Southeastern University	Florida	Health Informatics	Online		P		Use of informatics to improve health provider and healthcare system performance. Enhance wellness and disease prevention. Improve patient outcomes and reduce morbidity and mortality. Reduce medical errors and promote patient safety. Promote cost-effective healthcare. Facilitate the adoption of health information technology.
University of Pennsylvania School of Medicine	Pennsylvania	Biomedical Informatics	On-campus		P		Identify relevant information science and technology concepts, methods, and tools to solve health informatics problems. Apply social, behavioral, legal, psychological, management, cognitive, and economic theories to design, implement, and evaluate health informatics solutions. Choose appropriate biomedical and health information tools and methods to solve specific health information problems. Design solutions to health problems using computational thinking, information science, and technology. Understand the roles of various health professionals and stakeholders, including patients, and apply team science principles to address complex health issues.
Charter Oak State College	Connecticut	Health Informatics	Online		P		Interdisciplinary study of the design, development, adoption, and application of information, data and technology-driven innovations in healthcare.
University of Mississippi Medical Center	Mississippi	Health Informatics and Information Management	Online		P		Knowledge and skills in the areas of project management; databases and computer science; data analytics and visualization; human-computer interaction; information security; epidemiology and public health; and leadership.
SUNY Stony Brook University	New York	Applied Health Informatics	Online		P		Exposes students to the latest tools and techniques in data analytics. Provides knowledge in clinical information systems management. Covers project management skills necessary for technology management. Focuses on technologies used by health systems, payers, and technology companies. Aims to improve patient outcomes through effective technology management.
Oregon Health & Science University	Oregon	Biomedical Informatics	Hybrid		P	P	Use data to improve patient care, public health and biomedical research. Handle, analyze, and understand large amounts of data produced by the advanced techniques used in modern biological research.
University of Wisconsin-Madison	Wisconsin	Clinical & Health Informatics	Online		P		Leverage skills in operational and healthcare management, health informatics, and information technology to enhance professional practices. Make confident decisions regarding health, security, and biomedical and health information problems. Contribute to creating new solutions or improving existing ones for healthcare delivery, prevention, and outcomes.
Stanford University	California	Clinical Informatics Management/Biomedical Informatics	On-campus		P	P	Oversee and implement new technology in healthcare. Gain core business skills and insights into the health sector. Develop a management-focused and ethical understanding of digital innovations for diverse populations.
Carnegie Mellon University	Pennsylvania	Health Care Analytics & Information Technology	On-campus		P		Learn to convert raw data into actionable solutions for complex healthcare problems. Build the next generation of health technologies.
Idaho State University	Indiana	Health Informatics	Online		P		Transform data into knowledge to help healthcare providers and patients make informed medical decisions. Enhance health outcomes and advance medical research. Empower patients and enrich society.
Medical University of South Carolina	South Carolina	Health Informatics/Biomedical Informatics	Online and hybrid		P	P	Manage, use, and evaluate healthcare information systems, including EHRs, data management, and analytic systems. Gain firsthand experience working with experts in telehealth through MUSC’s Center of Telehealth.
UT Southwestern Medical Center	Texas	Health Informatics	On-campus		P		Provide students with interpersonal, cognitive, analytical, and applied skills needed to thrive in health informatics. Build on existing skills from previous education in medicine, health, computer science, or information technology. Gain knowledge from educators with firsthand experience of systems and system challenges. prepare graduates to initiate meaningful change and become multidisciplinary leaders in the field of health informatics.
University of Alabama at Birmingham	Alabama	Health Informatics	Online		P		Clinical, administrative, and health information systems, security in healthcare, strategic planning, and leadership development. Concentrate in data analytics with courses like Advanced Database Design, Business Intelligence for Healthcare, and Quantitative Methods; User experience with coursework in User-Based Design and User-Based Research, among other subjects. AI with courses such as Applications of AI in Medicine and Integration of Artificial Intelligence into Clinical Workflow.
East Carolina University	North Carolina	Health Informatics and Information Management	On-campus		P		Acquire, store, and use information in a healthcare setting. Collaborate with clinicians and health services administrators to develop clear and effective health information strategies for a healthcare organization.
University of Missouri	Missouri	Health Informatics	On-campus and hybrid		P	P	Students learn how information technology can enhance the integration, quality, and safety of clinical services. Emphasizes improving the efficiency and overall business functions of health systems. Design, develop, implement, and evaluate IT. prepares students to improve systems at both operational and enterprise levels.
University of Pittsburgh	Pennsylvania	Health Informatics	On-campus and online		P	P	Develop critical thinking skills to create data- and technology-driven informatics solutions. Utilize the most advanced technologies and industry best practices. Focus on improving patient care through innovative informatics strategies.
Massachusetts College of Pharmacy and Health Sciences	Massachusetts	Health Informatics	Online		P		Ensure ethical outcomes for patients, providers, and payers by applying compliance requirements. Identify barriers to care and build a more inclusive health system using data. Analyze electronic health records to support informed decision-making. Implement systems linking clinical data to healthcare providers and managers. Improve healthcare by analyzing patient data and clinical outcomes. Support teamwork and shared decision-making with data and informatics. Lead innovative solutions using informatics, data science, and technology.
University of Minnesota School of Nursing	Minnesota	Nursing Informatics	Hybrid		P		Link patients, providers, public health, and researchers through authorized and secure information sharing.
Columbia University Irving Medical Center	New York	Biomedical Informatics	On-campus		P	P	Core courses provide a foundation in biomedical informatics methods, techniques, and theories. Qualitative, quantitative, and IT objectives help students apply methods to areas like clinical informatics, clinical research, translational informatics, consumer health, and public health informatics. A specialization in data science is also offered.
University of Kansas Center for Health Informatics	Kansas	Health Informatics	Hybrid		P		Integrate health, social-behavioral, and informatics knowledge to improve the health and well-being of individuals and communities.
Vanderbilt University Medical Center	Tennessee	Applied Clinical Informatics	Hybrid		P	P	Address the specific informatics challenges facing your workplace. Analyze large data sets, including genomic, EHR, and consumer-driven data. Contribute to an information systems division or as chief information. Officer in a clinical setting. Pursue an AMIA health informatics certification. Join the next generation of clinical informatics leaders.
Case Western Reserve University	Ohio	Biomedical and Health Informatics	On-campus		P	P	Design studies and integrate large, complex data sets. Analyze data and manage projects and teams. Lead process improvements in clinical settings. Advance research impacting prevention and treatment.
University of Arkansas for Medical Sciences	Arkansas	Biomedical Informatics	On-campus		P	P	Enhance knowledge and skills in applying biomedical informatics principles and methods within clinical practice. Work effectively in an interdisciplinary team.
Northwestern University Feinberg School of Medicine	Illinois	Health Informatics	Online		P		Anticipate and assess evolving health informatics needs from clinical, technical, operational and financial perspectives. Create a vision for the use of information to improve the quality, safety, and efficiency of patient-centered care and public health. Nurture the development of leadership skills to navigate the privacy, security, legal, regulatory, ethical, and social challenges inherent to health informatics. Develop essential skills such as organizational change leadership and project management.
Kennesaw State University	Georgia	Healthcare Management and Informatics	Hybrid		P		Understand the connections between healthcare management and informatics. Recognize the importance of integrating these fields for effective 21^st^-century healthcare delivery. Essential for leadership in the evolving healthcare industry.
SUNY Downstate Health Sciences University	New York	Medical Informatics	On-campus		P		Organize, store, and retrieve health information data. Coursework includes health care delivery, network architecture, database systems, and medical imaging.
Washington University in St. Louis	Missouri	Biomedical Informatics	On-campus and hybrid		P	P	Provide students with competency-based training in core biomedical informatics theories and methods. Address the shift toward transdisciplinary, integrative, and data-intensive approaches in health and life sciences research.
University of Utah	Utah	Biomedical Informatics/Nursing Informatics	Hybrid		P	P	Train students in applied technical skills and management of health care analytics. Prepare students with analytic skills and knowledge to improve systems supporting clinical decision-making, bridge technology with patient care, and advance into leadership roles influencing systems of care.
Florida International University	Florida	Health & Analytics	Online		P		Prepare information systems professionals, healthcare managers, healthcare support personnel, physicians, nurses, and other clinicians to use health information to improve the quality, safety, outcomes, and cost-effectiveness of healthcare delivery.
Weill Cornell Medical College	New York	Health Informatics	On-campus		P		Train in using informatics to improve care quality. Learn about healthcare organization and delivery, standards and interoperability, health data management, environmental health informatics, clinical informatics, biostatistics, and healthcare transformation.
University of Florida Health	Florida	Biomedical Informatics	On-campus		P	P	Develop a theoretical and practical understanding of informatics-related topics in Biology, Healthcare, and public health. Apply knowledge to improve health-related outcomes.
The Ohio State University	Ohio	Biomedical Informatics	On-campus		P	P	Learn methodologies and tools for big data and biomolecular analyses, implementation science, and clinical informatics. Benefit from diverse faculty expertise in biomedical informatics research. Gain a strong background in public health, including coursework in biostatistics and epidemiology.
University at Buffalo School of Medicine and Biomedical Sciences	New York	Biomedical Informatics	Hybrid		P	P	Emphasizes self-study, collaboration, and problem-solving in bioinformatics and translational research informatics, clinical informatics and decision support, clinical population research and public health informatics, biomedical ontology, and sociotechnical and human-centered design.
University of Cincinnati	Ohio	Biomedical Informatics	On-campus			P	Offer a comprehensive understanding of essential analytical concepts in informatics and biomedical data science, from molecular levels to individuals and populations.
University of North Carolina at Chapel Hill	North Carolina	Health Informatics	Hybrid			P	Core and frontier topics in health informatics (foundational concepts of informatics). Tools and infrastructure (advanced techniques for manipulating health data). Research methods and execution of research. Project management and academic leadership skills. Implementation science and translation.
UCLA Health - David Geffen School of Medicine	California	Medical Informatics	On-campus			P	Cover foundational and contemporary topics in biomedical informatics, with a focus on (bio) statistics and data science applied to biomedical and clinical use cases. Research emphasizes the development, evaluation, and implementation of modern techniques in real-world healthcare settings.

Abbreviations: CDSS: Clinical decision support system; EHR: Electronic health record; IT: Information technology.

**Table 2.** Accreditation Council for Graduate Medical Education (ACGME)-accredited clinical informatics programs

Code	Program name	Location (city, state)

Clinical informatics (Family medicine, 8)		
220312001	HonorHealth Program	Scottsdale, Arizona
1222612001	University of Minnesota/University of Minnesota Medical Center Program	Minneapolis, Minnesota
1223614003	University of North Carolina Hospitals Program	Morrisville, North Carolina
1223812003	Kettering Health Network/Indu and Raj Soin Medical Center Program	Beavercreek, Ohio
1223814002	Ohio State University Hospital Program	Columbus, Ohio
1224812001	Waco Family Medicine - Institute Program	Waco, Texas
1225412001	Madigan Army Medical Center Program	Tacoma, Washington
1225412003	University of Washington Program	Seattle, Washington

Clinical informatics (Internal medicine, 32)		
1390314001	University of Arizona College of Medicine-Phoenix Program	Phoenix, Arizona
1390514001	UCLA David Geffen School of Medicine/UCLA Medical Center Program	Los Angeles, California
1390514002	University of California (San Francisco) Program	San Francisco, California
1390514003	Cedars-Sinai Medical Center Program	Los Angeles, California
1390514004	University of California (Irvine) Program	Orange, California
1390514005	University of California (San Diego) Medical Center Program	San Diego, California
1390800001	Yale-New Haven Medical Center Program	New Haven, Connecticut
1390914001	Sidney Kimmel Medical College at Thomas Jefferson University/Christiana Care Health Services Program	Wilmington, Delaware
1391114003	University of South Florida Morsani College of Medicine/Moffitt Cancer Center/Tampa General Hospital Program	Tampa, Florida
1391614002	Rush University Medical Center Program	Chicago, Illinois
1391700001	Indiana University School of Medicine Program	Indianapolis, Indiana
1391814001	University of Iowa Hospitals and Clinics Program	Iowa City, Iowa
1391914001	University of Kansas School of Medicine Program	Kansas City, Kansas
1392114001	Louisiana State University (Shreveport) Program	Shreveport, Louisiana
1392414001	Beth Israel Deaconess Medical Center Program	Boston, Massachusetts
1392414002	UMass Chan Medical School Program	Worcester, Massachusetts
1392614001	Hennepin Healthcare Program	Minneapolis, Minnesota
1393414001	University of New Mexico School of Medicine Program	Albuquerque, New Mexico
1393514001	Icahn School of Medicine at Mount Sinai Program	New York City, New York
1393514002	New York Presbyterian Hospital (Columbia Campus) Program	New York City, New York
1393514004	Rochester General Hospital Program	Rochester, New York
1393514005	NYU Grossman School of Medicine Program	New York City, New York
1393614002	Duke University Hospital Program	Durham, North Carolina
1393814001	The MetroHealth System/Case Western Reserve University Program	Cleveland, Ohio
1394014001	Oregon Health & Science University (OHSU Health) Program	Portland, Oregon
1394114001	UPMC Medical Education Program	Pittsburgh, Pennsylvania
1394114002	Geisinger Health System Program	Danville, Pennsylvania
1394514001	Prisma Health/University of South Carolina SOM Greenville (Greer) Program	Greer, South Carolina
1394704001	Vanderbilt University Medical Center Program	Nashville, Tennessee
1394800001	Baylor Scott and White Medical Center - Round Rock Program	Round Rock, Texas
1394814001	University of Texas Health Science Center at Houston Program	Houston, Texas
1395614001	University of Wisconsin Hospitals and Clinics Program	Madison, Wisconsin

Clinical informatics (Pathology, 10)		
3020530001	University of California Davis Health Program	Sacramento, California
3021630002	McGaw Medical Center of Northwestern University Program	Chicago, Illinois
3021638001	University of Illinois College of Medicine at Chicago Program	Chicago, Illinois
3022430001	Mass General Brigham/Massachusetts General Hospital/Brigham and Women's Hospital Program	Boston, Massachusetts
3022630001	Mayo Clinic College of Medicine and Science (Rochester) Program	Rochester, Minnesota
3023530001	University at Buffalo Program	Buffalo, New York
3023530002	Stony Brook Medicine/University Hospital Program	Stony Brook, New York
3024830001	Houston Methodist Hospital (Medical Center) Program	Houston, Texas
3024830002	University of Texas Health Science Center San Antonio Joe and Teresa Lozano Long School of Medicine Program	San Antonio, Texas
3024830005	Baylor College of Medicine Program	Houston, Texas

Clinical informatics (Pediatrics, 11)		
3220432001	University of Arkansas for Medical Sciences (UAMS) College of Medicine Program	Little Rock, Arkansas
3220504001	Stanford Health Care-Sponsored Stanford University Program	Palo Alto, California
3220532001	Children's Hospital Los Angeles Program	Los Angeles, California
3221232001	Emory University School of Medicine Program	Atlanta, Georgia
3221632001	University of Chicago Program	Chicago, Illinois
3222432001	Boston Children’s Hospital/Boston Medical Center Program	Boston, Massachusetts
3222832001	Washington University/B-JH/SLCH Consortium Program	St. Louis, Missouri
3223832002	Cincinnati Children’s Hospital Medical Center Program	Cincinnati, Ohio
3224132001	Children’s Hospital of Philadelphia Program	Philadelphia, Pennsylvania
3224832001	University of Texas Southwestern Medical Center Program	Dallas, Texas
3225132001	University of Virginia Medical Center Program	Charlottesville, Virginia

**Table 3.** Student background surveys at the Master’s (MS) level

Student professional background	Online MS class (*N*=33)		In-person MS class (*N*=54)	
	*N*	Percentage	*N*	Percentage

Pharmacy	14	42.4	6	11.1
Dentistry	4	12.1	5	9.3
Health informatics	4	12.1	2	3.7
Physician	2	6.0	3	5.6
Nursing	3	9.0	1	1.9
Radiologic technologist	1	3.0		
Nutrition	1	3.0		
Public health	1	3.0	1	1.9
Psychology	1	3.0		
Engineering			20	37.0
Computer science			7	13.0
Biology			1	1.9
Business			1	1.9
Missing	2	6.0	7	13.0

**Table 4.** Summary of challenges and proposed solutions in clinical informatics (CI) education

Challenges in CI	Our practice/solution	Lessons learned/remaining challenges

1. Scope and content	Based on textbooks, publications, competency requirements and our experience	Bridging existing competency and the actual topics/content in a course
2. Students with diverse professional backgrounds	Group projects, electives for pre-requisite, flexible/optional content, and extra or additional course materials	More effective teamwork in addition to discussion and projects
3. Comprehensive evaluation	Multi-level course evaluation about the content, delivery, learning outcomes, and rubrics	Possibility to generate rubrics to assess course content, delivery, and student’s learning outcomes
4. Continuous mentoring	Micro-(project) and Macro-(career, field) guidance, lifelong learning, bond students to promote peer mentoring/support	Effective strategy to engage and continue mentoring, connection, and support after graduation
5. New technology impacts	SWOT* analysis, comparison, systematic evaluation, and demonstration	Open mind, constructively leverage new technology in CI education

* SWOT: Strengths, weaknesses, opportunities, and threats.

## Appendix

### A1. Sample of a pre-course student survey

1. Do you have any clinical background?
  - Yes
  - No
2. Do you have any HIT background?
  - Yes
  - No
3. What kind of experience do you have with healthcare data?
  - None
  - Limited (e.g., basic understanding)
  - Moderate (e.g., work or academic experience)
  - Advanced (e.g., extensive professional experience)
4. Are you familiar with any CDSS?
  - Yes
  - No
5. What are your career goals after completing this class or this program?
  [Open-ended response]

### A2. A sample team project for a CDSS course

#### Assignment description

Each team can include 3 to 5 students with different backgrounds and prior experience: at least one student with a medical, nursing, or other healthcare-related background, one with a computer science or information system background, and one with a healthcare administrative background. The recommended time span for the project is 6–7 weeks (i.e., half semester). Each team is required to select two vaccines from the CDC-recommended vaccination schedule (e.g., MMR, polio, HPV, DTap, Tdap, https://www.cdc.gov/vaccines/schedules/hcp/imz/child-adolescent.html#table-1) to work on:

1. Translate two selected vaccines (birth to 15 months, regular schedule) into a tabular format – human-readable (week 1);
2. Translate the two selected vaccines into an algorithm description format – human-readable (week 2);
3. Use the AHRQ CDS Authoring tool (https://cds.ahrq.gov/authoring/) to prepare CQL (clinical quality language) files that could be executed by a CQL engine (https://ecqi.healthit.gov/tool/cql-evaluation-engine) (weeks 3–4);
4. Use NLM Value Set Authoring Center (https://vsac.nlm.nih.gov/) to represent the needed concepts in machine codes in CQL (weeks 3–4);
5. Test CQL files using a CQL engine and synthetic patients’ data in FHIR resources format (https://dataverse.harvard.edu/dataset.xhtml?persistentId=doi:10.7910/DVN/QDXLWR ) (weeks 5–7).
6. Revise CQL files if necessary (weeks 3–7).
7. Record the CQL file execution process and successful results and include the video as part of the presentation(weeks 6–7).
8. Write the paper (weeks 1–7).

#### Assignment objectives

This assignment aims to help students understand and obtain hands-on experience translating recommended rules into human-readable and machine-processable formats. A long process and multiple expertise are needed to achieve the goal. Any minor mistake can cause potential harm to patients, which can be used as a teachable moment to remind students of the complexity of patient safety and the potential role of HIT in the process. Meanwhile, let students understand that healthcare is teamwork and that all expertise should be appreciated and needed in order to provide safe and high-quality clinical care. In addition, let students be familiar with the healthcare data standards and their applications in the real world through the NLM Value Set Authoring Center.

#### For instructors

This course is designed for health/medical informatics Ph.D.-level students. Therefore, instructors can pick and select parts of the assignment to customize it to their students and teaching needs. Weekly or biweekly milestones can be used to facilitate students’ making anticipated progress, identifying problems, and seeking help as early as possible.

#### Assignment requirements

Students need to prepare a brief paper for the assignment. The paper should include:

1. A brief introduction of the topic within the context of the lecture, reading, and discussion in class;
2. A detailed description of the workflow and methods to conduct the assignment, including who is doing what for the project and how the team completes the assignment;
3. An analysis (pros, cons, strengths, weaknesses, etc) of the topic and your assignment;
4. A results section to report your findings;
5. A discussion section includes
  - The reflections on the project, how the project is relevant to patient safety,
  - What would be the potential harm to patients if there are any errors in the process,
  - The limitations of the solution, the challenges of using HIT to keep patients safe,
  - A proposed and improved plan for future work,
  - How the proposed plan may benefit patient safety in clinical care.
  - WHAT are the most important lessons you have learned through this project and
6. Conclusion.
  The paper should be at least 2000 words, excluding tables or figures, with brief structures abstract (<250 words, including background, methods, results, and conclusion) and keywords. Students are strongly encouraged to use tables and graphs whenever appropriate. All tables and figures should have a number and a title and should be cited in the writing first. All references should be cited numerically and listed in Vancouver Style (e.g., JAMA references style). Students are encouraged to use examples or cases to make the writing more specific. The presentation should last 10 – 15 minutes and include slides, examples, data, and references.

#### For students

All assignments should be prepared from scratch; no AI generative texts should be used. All papers should be submitted via TurnItIn for plagiarism and AI generative text checks.

Students are encouraged to use weekly milestones to make progress.

Most importantly, students need to determine the following before starting to work on the project:

1. how to split the tasks and
2. assign tasks to each team member, who will do which task, and prepare the deliverables, i.e., paper, recordings, and presentation
3. what would be the regular team meetings day/time
4. how the team will communicate with each other
5. how the collaboration will take place
6. be respectful, be responsible, be accountable, and be a good team player.

### A3. A use case for enhancing clinical decision support with OpenEMR

#### Scenario

Students are tasked with modifying CDS rules within OpenEMR, an open-source EHR solution, to enhance patient care and workflow efficiency at a healthcare organization.

#### Objective

To leverage OpenEMR’s capabilities to customize CDS rules, ensuring that clinicians receive timely and relevant information tailored to their specific workflows and patient populations.

#### Steps

##### Understanding OpenEMR’s Capabilities

Students explore OpenEMR’s features, including patient scheduling, billing, and e-prescribing, its flexibility for CDS rule modification, interoperability with other healthcare systems with supporting standards like HL7, and comprehensive functionality for managing patient data.

##### Assessing Healthcare Organization’s Needs

Students collaborate with clinicians and healthcare administrators to identify key areas where CDS rule modification can improve patient care and operational efficiency.

##### Customizing CDS Rules

Using OpenEMR’s built-in tools and interfaces to modify CDS rules to align with the identified needs and workflows of the healthcare organization.

Students configure rules to trigger alerts, reminders, or clinical guidelines based on predefined criteria such as patient demographics, medical history, and clinical indicators.

##### Testing and Validation

Students conduct thorough testing and validation of the modified CDS rules within a controlled environment to ensure accuracy, reliability, and usability.

### A4. A sample rubric for a project peer evaluation

Peer assessment rubric for group projects

Name:

This form is an assessment for (student name: )

Group #

Project #

Date:

**Table:**

	0 – 2	3 – 5	6 – 8	9 – 10	Points

Contribution					
Research and gather information	Do not collect any information that relates to the topic.	Collects very little information-- some relates to the topic.	Collects some basic information--most relates to the topic.	Collect a great deal of information-- all relates to the topic.	
Share information	Does not relay any information to teammates.	Relays very little information-- some relate to the topic.	Relays some basic information--most relates to the topic.	Relays a great deal of information-- all relate to the topic.	
Be punctual	Does not hand in any assignments.	Hands in most assignments late.	Hands in most assignments on time.	Hands in all assignments on time.	
Take responsibility					
Fulfill team role’s duties	Does not perform any duties of assigned team role.	Performs very little duties.	Performs nearly all duties.	Performs all duties of assigned team roles.	
Participate in group meetings	Does not speak during the meetings.	Either gives too little information or information which is irrelevant to the topic.	Offers some information--most is relevant.	Offers a fair amount of important information-- all is relevant.	
Share equally	Always relies on others to do the work.	Rarely does the assigned work-- often needs reminding.	Usually does the assigned work-- rarely needs reminding.	Always does the assigned work without having to be reminded.	

	0 – 2	3 – 5	6 – 8	9 – 10	Points

Value others’ viewpoints					
Listen to other teammates	Is always talking-- never allows anyone else to speak.	Usually doing most of the talking-- rarely allows others to speak.	Listens, but sometimes talks too much.	Listens and speaks a fair amount.	
Cooperate with teammates	Usually argues with teammates.	Sometimes argues.	Rarely argues.	Never argues with teammates.	
Make fair decisions	Usually wants to have things their way.	Often sides with friends instead of considering all views.	Usually considers all views.	Always helps the team to reach a fair decision.	
Total					

Reference: http://edweb.sdsu.edu/triton/tidepoolunit/Rubrics/collrubric.html

### A5. A sample rubric for a project evaluation

Project assessment rubric

Student name:

Project #

Date:

**Table:**

Criteria					Points
	0 – 2	3 – 5	6 – 8	9 – 10	

Abstract	Does not fully explain the point of the paper; exceeds or has a significantly smaller required word limit.	Partially explains the point of the paper; exceeds or has a significantly smaller required word limit.	Adequately explains the point of the paper; meets or nearly meets word limit.	Fully explains the point of the paper; meets word limit.	
Content	Lacks depth, breadth, or understanding of basic facts or concepts.	Shows adequate understanding of basic facts or concepts.	Shows proficient understanding of basic facts or concepts; shows basic application, analysis, and synthesis capabilities.	Shows extensive understanding of basic facts or concepts; shows advanced application, analysis, and synthesis capabilities.	
Organization	The content is listed without internal connections and lacks of focus.	The content does not flow smoothly and logically.	The content is unified, focused but not well organized.	The content is organized well, unified, focused and it flows naturally.	
Tables and graphics	Inappropriate tables or graphics.	Few or no tables or graphics when using them can enhance the content.	Two or more appropriate tables and graphics.	Uses 2 or more tables and graphics to present clearer and straight forward content.	
Conclusions reached	A conclusion is made from the evidence offered.	Some detailed conclusions are reached from the evidence offered.	Several detailed and clear conclusions are reached from the evidence offered.	Several detailed, clearer, solid and constructive conclusions are reached from the evidence offered.	
References and literature review	Information gathered is inadequate, outdated, or improperly cited.	Information is gathered from limited electronic and non-electronic sources; some references are not properly cited.	Information is gathered from extensive electronic and non-electronic sources; few references are not properly cited.	Information is gathered from extensive electronic and non-electronic sources; all references are properly cited.	
Citations	More than two mistakes in citations’ numeric order or inconsistent style.	Two mistakes in citations’ numeric order or inconsistent style.	One mistake in citation’s numeric order or inconsistent style.	All the citations are listed in numeric order and in a consistent style.	
Formatting	Does not follow proper formatting; numerous errors in spelling, grammar or punctuation.	Does not follow proper formatting; few errors in spelling, grammar or punctuation.	Properly formatted; few errors in spelling, grammar or punctuation.	Properly formatted; no errors in spelling, grammar or punctuation.	
Oral presentation	Great difficulty communicating ideas. Poor voice projection. Little preparation or incomplete work.	Some difficulty communicating ideas, due to voice projection, lack of preparation, or incomplete work.	Communicates ideas with proper voice projection. Adequate preparation and delivery.	Communicates ideas with enthusiasm, proper voice projection, appropriate language, and clear delivery.	
Total					

Reference: Scientist teaching science training material; http://www.ncsu.edu/midlink/rub.mmproj.htm
